# 712 Outcomes of Transplants with CEA Derived from a Modified Composite Culture Technique: A Case Series

**DOI:** 10.1093/jbcr/irae036.256

**Published:** 2024-04-17

**Authors:** Wayne G Kleintjes, Tarryn K Prinsloo

**Affiliations:** Stellenbosch University, Cape Town, Western Cape; Cape Peninsula University of Technology/Biosigns Therapeutics, Cape Town, Western Cape; Stellenbosch University, Cape Town, Western Cape; Cape Peninsula University of Technology/Biosigns Therapeutics, Cape Town, Western Cape

## Abstract

**Introduction:**

Establishing low-cost and efficient culture environments comparable to standard techniques would undoubtedly improve technique usage in under-resourced settings. The aim of this prospective, cohort case series was to report on the CEA graft-take using a modified culture technique that could benefit under-resourced clinical environments in terms of CEA accessibility and expense.

**Methods:**

Burn patients (n=25) with low survival prognosis, large total body surface area (TBSA), and/or exhausted donor sites were transplanted with CEA grown on routinely used hydrophobic gauze in a pediatric incubator. Transplants occurred at our specialized Burns Centre between October 2014 and March 2016, following emergency ethical approval. Keratinocytes were retrieved from 3 x 2 cm full-thickness skin biopsies, placed in trypsin solution, and seeded onto the gauze. The cells were incubated in pediatric incubators at 37 °C and fresh autogenous plasma was applied daily while Intrasite gel® every third or fourth day until confluency on day 14 was reached. CEA was transplanted onto the debrided wound beds. Xenografts were used for temporary cover during the culture period. Graft take was assessed on burns patients that survived after the CEA transplant for ≥ 21 days (final assessment). The primary outcome was graft take, calculated as a surface area percentage of skin graft to CEA and the secondary outcome was main complications (nutrition, perineum burns, and hypoxic brain). Central indices were described as mean (95% CI) and/or frequency (%) for the following parameters: age, %TBSA, abbreviated burn severity index (ABSI) scores, % survival relative to ABSI and % graft take.

**Results:**

Eleven patients (44%) survived until the final assessment (mortality occurred during the culture period), however, one case was excluded since treatment was for cosmetic purposes. Of the transplant cases, 10 out of 11 patients survived. A mean age of 36.1 years (95% CI 25.8-46.4), 45.0 %TBSA burns (95% CI 35.1-54.9), 9.7 ABSI scores (95% CI 8.6-10.8) and 79.5% graft take (95% CI) was observed. Reduced graft take (61.2%) was observed in patients with perineum burns compared to those with the remaining reported complications (91.8%).

**Conclusions:**

CEA graft take was 79.5% using a low-cost culture technique was comparable to the largest CEA-based case series in literature. The survival of the major burn cases was highly favourable considering the severity of injuries, expected outcomes without CEA and the reported challenges.

**Applicability of Research to Practice:**

Consistent graft take with a reliable, low-cost CEA method has been elusive until this method was applied in our setting. There is a potential for this method to be applicable in other settings as well and therefore make a value contribution to the accessibility of CEAs in general.

